# Crosstalk between Biomolecular Condensates and Proteostasis

**DOI:** 10.3390/cells11152415

**Published:** 2022-08-04

**Authors:** Emmanuel Amzallag, Eran Hornstein

**Affiliations:** 1 Department of Molecular Neuroscience, Weizmann Institute of Science, Rehovot 7610001, Israel; 2 Department of Molecular Genetics, Weizmann Institute of Science, Rehovot 7610001, Israel

**Keywords:** biomolecular condensation, amyotrophic lateral sclerosis, ALS, neurodegeneration, proteostasis, membraneless organelles, ubiquitin proteasome system

## Abstract

Proper homeostasis of the proteome, referred to as proteostasis, is maintained by chaperone-dependent refolding of misfolded proteins and by protein degradation via the ubiquitin-proteasome system and the autophagic machinery. This review will discuss a crosstalk between biomolecular condensates and proteostasis, whereby the crowding of proteostasis factors into macromolecular assemblies is often established by phase separation of membraneless biomolecular condensates. Specifically, ubiquitin and other posttranslational modifications come into play as agents of phase separation, essential for the formation of condensates and for ubiquitin-proteasome system activity. Furthermore, an intriguing connection associates malfunction of the same pathways to the accumulation of misfolded and ubiquitinated proteins in aberrant condensates, the formation of protein aggregates, and finally, to the pathogenesis of neurodegenerative diseases. The crosstalk between biomolecular condensates and proteostasis is an emerging theme in cellular and disease biology and further studies will focus on delineating specific molecular pathways involved in the pathogenesis of amyotrophic lateral sclerosis (ALS) and other neurodegenerative diseases.

## 1. Crosstalk between Biomolecular Condensates and Proteostasis

Proteostasis is the term given to the cellular systems, which maintain the proteome in a functional state. Proteostasis relies on the activity of chaperones that mediate protein conformational refolding and repair and on the protein degradation machinery, comprised of the ubiquitin-proteasome system (UPS) and autophagy. Cellular stress and aging both cause a decrease in proteostasis capacity which contributes to the pathogenesis of neurodegenerative diseases associated with protein aggregation [1,2]. Both intrinsic properties of a protein’s primary sequence and its cellular abundance are associated with its aggregation propensity. A subset of the proteome has been discovered to exhibit a high expression level relative to solubility [3,4,5], and these proteins were found to associate with insoluble inclusions in a range of neurodegenerative diseases, including Alzheimer’s amyloid plaques, Lewy bodies, and ALS inclusions [3,4]. These so-called supersaturated proteins require constant activity of proteostasis machinery to maintain a soluble, non-aggregated state [6,7,8]. Thus the joint activity of the cell’s proteostatic systems keeps the levels of misfolded proteins sufficiently low to prevent the formation of cytotoxic protein aggregates [9,10].

Biomolecular condensates are macromolecular assemblies that are not encapsulated by a phospholipid bilayer and are hence also referred to as membraneless organelles. Condensates enable the compartmentalization and crowding of defined biomolecules that promote the activity of specific enzymatic processes. Condensation obeys basic biophysical principles of liquid–liquid phase separation (LLPS) of the biomolecules inside the condensate from the surrounding liquid, often cytoplasm or nucleoplasm [11,12,13]. Multivalent interactions between the biomolecules are key to phase separation. Valency in this context refers to a property which describes the number of binding sites within a molecule to which other molecules may attach. Multivalent proteins drive phase separation by interacting with multiple binding partners, leading to the formation of membraneless condensates [12]. The regulation of multivalent interactions and their specificity establishes particular compositions in defined condensates, recruiting some biomolecules and rejecting others.

In this review, we describe the emerging crosstalk between biomolecular condensates and proteostasis, with implications to cellular and disease biology.

## 2. An Emerging Family of Ubiquitin Condensates

### 2.1. The Ubiquitin-Proteasome System

Ubiquitination is an endogenous post-translational protein-tagging system, whereby ubiquitinated proteins are marked for degradation by the proteasome, establishing the ubiquitin-proteasome system (UPS) [14,15]. In addition to proteasomal degradation, ubiquitinated proteins may also be directed to degradation via the autophagic-lysosomal pathway [14,16]. Mechanistically, successive activity of ubiquitin-activating and -conjugating enzymes (E1, E2, and E3 families) transfer ubiquitin to lysine residues on target proteins. Proteins destined for degradation are polyubiquitinated with chains of ubiquitin, which are linked to each other via a specific lysine residue. Importantly, the different linkage types differ in their 3D conformation and are thus recognized by different sets of protein interactors, which direct the target proteins to different cellular processes [14,17]. Canonically, K48-linked polyubiquitin chains direct target proteins towards proteasomal degradation, while K63-linked polyubiquitin chains direct proteins to lysosomal degradation [14,17]. This is followed by engagement of specialized ubiquitin-binding shuttle proteins, including ubiquillins, RAD23B and p62, that bind ubiquitinated proteins and introduce them to the proteasome where they are broken down. However, only recently has it become apparent that the recruitment of UPS components to specialized membraneless condensates is an important biochemical feature of proper UPS function. Interestingly, recent studies have demonstrated the concept that polyubiquitin chains can function as multivalent molecules, which can drive either the assembly or the disassembly of condensates via interactions with various ubiquitin-binding proteins.

### 2.2. Ubiquitin-Binding Shuttle Proteins Drive Formation of Nuclear Condensates

Although the proteasome complex itself has ubiquitin-binding subunits, efficient delivery of ubiquitinated substrates to the proteasome is mediated by ubiquitin-binding shuttle proteins [18]. Such proteins are conserved across all eukaryotes and are characterized by containing at least one ubiquitin-associating (UBA) domain, which binds ubiquitin, and a ubiquitin-like (UBL) domain which binds proteasomes [18]. RAD23B is a typical ubiquitin-binding shuttle protein consisting of two UBA domains on its C-terminal and an N-terminal UBL domain. Other ubiquitin-binding shuttles exhibit additional structural characteristics which impact their function. The ubiquitin-binding protein p62 contains a single C-terminal UBA domain, however it exhibits oligomerization capacity dependent on its PB1 domain, which is crucial to its function in delivering ubiquitinated substrates to both the proteasome and to the autophagic machinery [19,20,21,22,23]. Ubiquilin 2 (UBQLN2) interacts with HSP70 via its ST1-like domains and contains an intrinsically disordered domain [24,25]. Thus, ubiquitin-binding shuttle proteins are crucial components of the cellular protein degradation machinery, which also have important distinct structural characteristics that regulate their function.

The cellular stress response initiates the assembly of transient condensates in the cytoplasm and the nucleus. Specific nuclear condensates, which contain active proteasomes and polyubiquitinated proteins, have been recently described [26]. These nuclear proteasomal condensates exhibit characteristics of liquid droplets, typical of phase-separated liquids, and depend on the presence of ubiquitinated proteins. The ubiquitin-binding shuttle protein RAD23B is pivotal in the formation of this type of nuclear proteasomal condensates. RAD23B condensation depends on its ability to bind ubiquitinated substrates via its two UBA domains [27]. Furthermore, condensates were found to include entire proteasomes, which are directly recruited by RAD23B via its N-terminal ubiquitin-like (UBL) domain, and to contain the E3 ligase E6-AP and the ubiquitin-binding chaperone VCP. RAD23B condensates may function in placing substrates, enzymes and the degradation machinery in proximity to each other, thus improving local concentrations and efficiency in response to cellular stress.

Clearance of RAD23B-positive nuclear proteasomal condensates is dependent on proteasomal and VCP activity, suggesting that degradation of ubiquitinated substrates within the condensates is required for cellular recovery from stress and is necessary for condensate dissolution [26]. Accordingly, inhibition of either proteasomal or VCP activities results in accumulation of ubiquitinated substrates within these nuclear condensates, illustrating that insufficient proteostasis can result in the formation of persistent protein aggregates. This is supported by the finding that a loss of RAD23B function results in agenesis of the corresponding nuclear condensates and in apoptosis. Thus, the formation of RAD23B-positive condensates safeguards cells against apoptosis and demonstrates that UPS activity in defined condensates is an adaptive means for cells to overcome proteotoxic stress.

**Figure 1:**
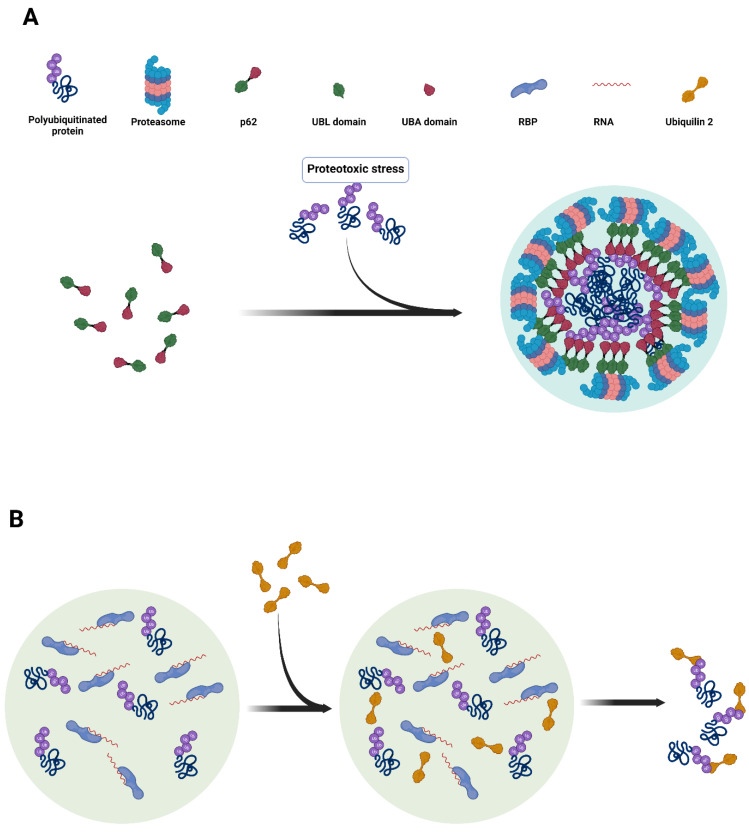
(**A**) Formation of nuclear p62 UPS condensates. Proteotoxic stress causes an increase in the number of ubiquitinated substrates, which require degradation. Polyubiquitin chains function as multivalent molecules, which drive condensation by binding to the UBA domain of p62. Oligomerization of p62 further contributes to multivalency. p62 recruits proteasomes to the condensate via its UBL domain. A similar mechanism drives the formation of RAD23B condensates, with the exception that RAD23B is not known to form oligomers like p62. (**B**) Model for UBQLN2-mediated extraction of ubiquitinated proteins from cytoplasmic SGs, consistent with the one proposed by [32]. SGs assemble via multivalent interactions between RNA-binding proteins and RNA. UBQLN2 enters SGs via its intrinsic phase separation propensity for phase separation. Once in SGs, UBQLN2 binding to ubiquitin chains causes it to dissociate from SGs, carrying with it bound ubiquitin-conjugated proteins. These are then likely delivered to the proteasome for degradation.

Other nuclear or cytoplasmic condensates are formed around another ubiquitin-binding shuttle protein, p62/SQSTM1, along with ubiquitinated substrates [19]. p62 condensates form through phase separation, whereby valency increases once the UBA domain binds polyubiquitinated proteins (Figure 1). Several domains within p62 contribute to its condensation propensity, including the PB1 oligomerization domain, weak electrostatic interactions of p62′s intrinsically disordered domain, and the LC3-interacting region (LIR) [20,21,22]. The function of cytoplasmic p62 condensates is to initiate degradation of the ubiquitinated proteins inside autophagosomes [23,28,29]. In this context, p62 condensation together with ubiquitinated substrates function as a platform upon which the autophagosomal membrane forms around the ubiquitinated cargo [23,28,29]. LC3, a critical component of the autophagosomal membrane, is recruited via the p62 LC3-interacting region (LIR). Interestingly, the LIR also plays a role in p62 condensation since its mutation results in decreased p62 condensate formation both in vitro and in vivo [23]. This dual effect of the LIR on LC3 recruitment and in p62 condensation suggests an intriguing mechanism whereby the LIR may act as a regulatory switch between the recruitment of autophagosomal cargo into dense condensates and its subsequent engulfment by the autophagosomal membrane, which forms upon LC3 binding to the p62 LIR [23]. In contrast to the role of cytoplasmic p62 condensates in the autophagic process, nuclear p62 condensates function as sites of active UPS activity, recruiting active proteasomes to initiate the degradation of ubiquitinated substrates in a manner analogous to the RAD23B condensates described above [19].

In another case, UBQLN2, a ubiquitin-binding shuttle that promotes degradation of ubiquitinated protein substrates in the proteasome [25], forms condensates in the cell’s nucleus and cytoplasm [25,30,31,32,33]. UBQLN2 is crucial to the cellular response to proteotoxic stress and loss of UBQLN2 results in increased cell death [25]. However, as opposed to p62 and RAD23B condensates, UBQLN2-positive condensates disassemble in response to the addition of ubiquitin chains [32], suggesting a low threshold of saturation and competitive inhibition by ubiquitin chains (Figure 1) [20,21]. In addition, NF-κB essential modulator (NEMO) forms condensates via multivalent interactions with polyubiquitin chains. The formation of these condensates is required for activation of the IκB kinase (IKK) complex upstream of NF-κB signaling [34,35].

Finally, the nuclear speckle (NS)-localized E3 ligase SPOP [36,37,38] has been shown to form nuclear condensates (distinct from NS) in cells when coexpressed with model substrates [39,40,41] and also forms liquid droplets in vitro [41]. Importantly, mutation of the SPOP oligomerization domains inhibit its incorporation into nuclear condensates in cells and prevents its ability to undergo LLPS in vitro, demonstrating that SPOP oligomers function as multivalent biomolecular entities which promote condensation [39,41]. Consistent with the proposed function of p62 and RAD23B nuclear condensates, SPOP nuclear condensates function as sites of active ubiquitination [41], and decreasing SPOP valency by mutation of the oligomerization domains reduces its ubiquitination efficiency [39,41]. It has been proposed that NS may function as sites of storage for SPOP when substrate levels are low, whereby increased substrate levels during cellular stress results in dissociation of SPOP from NS and condensation together with substrates into phase-separated condensates in which efficient ubiquitination occurs [41].

### 2.3. Additional Aspects of Multivalent Interactions in Ubiquitin Condensates

Multivalent interactions between ubiquitin-binding shuttle proteins (e.g., RAD23B or p62) and ubiquitinated peptides display additional layers of complexity: longer polyubiquitin chains provide more binding sites and thus increase valency [19,26]. Importantly, the relative concentrations of ubiquitin-binding shuttle proteins and ubiquitin chains dictate whether the system will condense or will remain soluble. This notion is illustrated by the effect of polyubiquitin chains on the morphology of p62 oligomers. At a low ubiquitin:p62 ratio, p62 assembles into round assemblies while saturating ubiquitin concentrations result in complete disassembly of p62 clusters, an effect which depends on the p62 UBA domain [21]. A lower ubiquitin:p62 ratio promotes the clustering of p62 by allowing each ubiquitin chain to preferentially interact with multiple p62 molecules. However, too many polyubiquitin molecules will instead favor interaction with a few p62 molecules, preventing condensation and effectively reducing the valency of polyubiquitin chains with respect to its interaction with p62 [20].

Concentration-dependent interplay between two components in control of phase behavior is essentially similar to the effect of RNA:protein ratio on valency [20,42,43,44] and illustrates that optimization of relative constituent concentrations governs condensate behavior.

Finally, the ubiquitin chain linkage type regulates condensate specificity and function. RAD23 condensates primarily contains Lysine 48 (K48), whereby the ubiquitinated proteins are often routed to proteasomal degradation [19,26,29,45,46]. In contrast, p62 nuclear condensates predominantly contain Lysine 63 (K63). K63 Ubiquitin chains undergo more efficient phase separation and are associated with lysosomal/autophagosomal protein degradation [17]. K63 ubiquitin chains are also the drivers of NEMO condensation, in which IKK is activated [34,35].

Together, it becomes clear that UPS activity preferentially appears in condensates. Condensation increases the efficiency of the UPS, which is critical for coping with the load of proteasomal ubiquitinated substrates under stressful cellular conditions. UBQLN2, p62, NEMO, and RAD23B all induce condensation behavior through binding of ubiquitinated proteins. The underlying biochemical principle is that propensity to condense, conveyed by multivalency, enables molecular crowding, and facilitates specific enzymatic activities. The existence of distinct condensates, with different molecular compositions and at specific cellular locations, imply that they might address related but separate properties and cellular needs.

These findings allude to the existence of a new family of membraneless condensates, in which ubiquitin conjugation and proteasomal degradation are coupled in high proximity within the same compartment. The existence of E1-E2-E3 cascade components, as described in the p62 condensates, imply higher ubiquitination processivity, and may be found also in other (e.g., RAD23B-positive) condensates. In addition, the internal organization of p62 nuclear condensates comprising an internal core of polyubiquitinated proteins that are surrounded by a p62-positive phase and an outer shell containing active proteasomes is intriguing and can be sought in other UPS-associated organelles. Such multi-phased condensates have been described, including stress granules [47,48,49] and the nucleolus [47], and may reveal shared principles in condensate biology for controlled successive steps. In the case of UPS condensates, this may be to allow stepwise ubiquitin conjugation and execution of proteasomal degradation.

## 3. Molecular Mechanisms of Proteostasis Converge on Cytoplasmic Stress Granules

### 3.1. Stress Granules and the Integrated Stress Response

Upon exposure to a variety of proteotoxic stressors, mammalian cells activate an adaptive cellular response known as the integrated stress response (ISR) to allow coping and recovery from the stressful conditions. The ISR involves a specific signal via any of four specialized kinases (PKR, PERK, GCN2, or HRI), which phosphorylate the translation-initiation factor eIF2a, at serine 51. This specific phosphorylation of eIF2a arrests protein translation, while concurrently promoting the selective translation of certain 5′ upstream ORF-bearing transcripts, which culminates in the activation of a network of key genes involved in proteostatic maintenance [50]. The ISR, via eIF2a phosphorylation, also involves the assembly of SGs. Akin to other condensates, stress granule (SG) condensation is driven by multivalent molecular interactions between RNA-binding proteins (RBPs) and non-translating mRNA. Weak electrostatic interactions between intrinsically disordered regions of the proteins further contribute to valency and condensation. Importantly, SGs swiftly dissolve back into the cytoplasm upon recovery from the stressful stimulus [51,52,53].

The protein GAP SH3 Domain-Binding Protein 1 (G3BP1) is the key driver of stress granule condensation and has been shown to reside in pre-stress granule complexes, under unstressed conditions [48,54,55,56]. Translation initiation complex proteins and dozens of other RBPs incorporate into the condensates and further drive their assembly. Furthermore, several RBPs which are involved in nuclear mRNA biogenesis processes, translocate into the cytoplasm under stress, where they are incorporated into SGs. The associated nuclear depletion contributes to the global reduction in protein synthesis [57].

### 3.2. Proteostasis Factors Regulate SG Dynamics

Ubiquitin has been shown to accumulate in SGs upon proteotoxic stress [58,59,60,61], however the potential role of the UPS in SGs is not well-understood. Polyubiquitinated proteins have been detected in SGs, a substantial fraction of which likely represents defective ribosomal products (DRiPs) which are nascent polypeptides prone to aggregation [61,62]. Some evidence suggests that other misfolding-prone polyubiquitinated proteins accumulate into SGs as well [63,64], but this is not yet conclusive and whether active ubiquitination takes place inside these condensates remains to be determined. It is possible that misfolded proteins are actively directed into SGs via specific cellular processes, such as by the activity of HSP70/HSP40 chaperones [65], or that misfolded proteins exhibit a passive propensity to accumulate into condensates, thereby carrying polyubiquitin chains with them. Whatever the molecular mechanism is, sequestration of misfolded proteins inside SGs may mitigate aggregation or contain it during times of proteotoxic stress. Accordingly, excess of misfolded proteins or insufficiency of refolding or degradation pathways may lead to excessive accumulation of misfolded proteins within SGs and derail their normal liquidity, driving them towards irreversible aggregation [62,63,66].

A few mechanisms have been described, by which proteostasis regulators contribute to SG dynamics. First, several molecular chaperones are recruited to SGs and are required for proper SG dynamics. DnaJ heat-shock protein 40 kD (Hsp40) interact with HSP70, and these co-chaperones contribute to SG assembly/disassembly dynamics [62,63,67,68,69,70]. The HSP70-HSP40 complex functions both in regulation of SG RBP condensation [67,69] but also takes part in dealing with the load of misfolded proteins accumulating in SGs [62,63]. This is likely by refolding activity and by recruiting the E3 ligase CHIP to mark irreversibly damaged proteins for proteasomal degradation [71].

### 3.3. Ubiquitin Signaling in SGs

The ubiquitin-selective segregase VCP/p97, which extracts ubiquitinated substrates from protein complexes, is crucial for SG disassembly [58,60,72,73,74]. Ubiquitination may also function as a signal for the VCP-mediated removal of specific SG proteins during condensate demixing. In support of this, a recent report demonstrated that VCP is involved in the extraction of K63-polyubiquitinated G3BP1 from SGs as a part of their disassembly [58], suggesting precise control of a specific key protein by ubiquitin conjugation in contrast to the mass action of weak multivalent interactions in p62 or RAD23B condensates.

E1 ubiquitin-activating enzyme is also critical for SG disassembly. However, some types of stressors induce SGs that are largely devoid of polyubiquitin and relatively resilient to inhibition of E1 ubiquitin-activating enzyme (e.g., sodium arsenite) [59,60,63,75,76,77], whereas polyubiquitination is evident with other stress cues, such as heat shock (HS). Accordingly, the disassembly of HS-initiated SGs is sensitive to inhibition of E1 ubiquitin activity [58,59,60,63,75]. A clue for the regulatory mechanism acting in SGs to degrade ubiquitinated substrates may be associated with UBQLN2. Thus, UBQLN2 is recruited into SGs where it binds to misfolded ubiquitinated substrates. In an elegant autoregulation mechanism, the binding of ubiquitinated substrates causes UBQLN2 to dissociate from SGs back into the cytoplasm, directing its bound substrates to degradation [20,32,78]. It is interesting to hypothesize that SGs’ liquid-like environment is a niche that is biochemically favorable for handling UPS and also HSP70-DNAJ client proteins [79]. Furthermore, ubiquitin conjugation and an autophagic flux are required for degradation of misfolded proteins. Insufficiency in these proteostatic mechanisms may result in ubiquitinated protein accumulation inside SGs and transition into a persistent aberrant state. 

### 3.4. Linking SUMO and StUbL to SGs

Small Ubiquitin-like Modifier (SUMO) is a ubiquitin-like moiety that is critical for SG dynamics. SUMO is necessary for SG assembly, presumably via increasing valency by producing interaction space for SUMO-conjugated peptides with SUMO interacting domains (SUMO glue model). This “glue-like” mechanism has been well described for promyelocytic leukemia (PML) protein nuclear bodies (NBs) where SUMOylation has been shown to drive the assembly and maturation of PML NBs by recruitment of SUMO interacting motif (SIM)-containing proteins [80,81]. A similar role for SUMOylation was recently reported in SG dynamics [48]. In addition, SUMOylation regulates disassembly by recruitment of the SUMO-targeted ubiquitin ligase (StUbL) pathway. StUbL results in ubiquitination of SUMOylated substrates by the E3 ubiquitin ligase RNF4 [82,83] and downstream proteasomal degradation. This mechanism is known to be critically involved in disassembly of PML NBs, where recruitment of the StUbL RNF4 results in degradation of PML protein [84,85]. In SGs, it is interesting to speculate that the dual SUMO function in SG assembly and disassembly, is biochemically coded by a transition from monoSUMOylation to polySUMOylation, but this remains to be explicitly tested experimentally [82]. SUMO-triggered and RNF4-mediated ubiquitination in both SGs and PML nuclear bodies reveal shared molecular mechanisms that govern the regulation of different condensates and expand the impact of ubiquitin-related post-translational modifications on condensate biology.

## 4. Stress Granules and Neuronal Inclusions in Amyotrophic Lateral Sclerosis

### 4.1. ALS and Cellular Proteostasis

Amyotrophic lateral sclerosis (ALS) is a neurodegenerative syndrome of the human motor neuron system, which resides on a clinical, genetic and pathology continuum with frontotemporal dementia (FTD), a form of early onset dementia. The major neuropathological hallmark of a human brain from a patient suffering from ALS and/or FTD, is the presence of ubiquitinated and hyper phosphorylated TDP-43 in neuronal inclusions [86]. Other RBPs have been detected in neurodegeneration-associated brain inclusion as well, although more variably. Furthermore, cellular proteostasis components such as UBQLN2, p62, OPTN, heat shock proteins and VCP were found in ALS-associated inclusions [30,87,88,89,90,91,92,93,94,95,96,97]. These observations associate altered proteostasis capacity with ALS pathogenesis. 

### 4.2. Aberrant SG Condensation May Lead to ALS Inclusion Formation

SGs make an intriguing cellular model for inclusions and may therefore be informative about mechanisms related to inclusion formation in the diseased brain [98,99,100,101]. Failed proteostasis causes the accumulation of damaged/misfolded proteins into SGs leading to aberrant condensation [62,63,66] and may be related to factors such as HSP70, VCP or UBQLN2 that localize to SGs [32,58,62,63,74]. In addition, several SG resident proteins are identified in inclusions [102,103,104], or are encoded by genes that are associated with ALS that are associated with ALS.

Finally, the ALS-associated hexanucleotide repeat expansion in C9ORF72, which is the most common genetic cause of ALS and FTD, provides several connections to SG biology: loss of normal C9ORF72 function impairs the interaction with p62 and normal autophagy [76], pathological dipeptide proteins that are generated in C9ORF72 disease incorporate into SGs, form cytotoxic aggregates and impair normal SG dynamics [48,105,106], in part by induction of ER stress [107,108] and in part by sequestering the proteasome [92,93]. 

### 4.3. Central Proteostasis Pathways Regulate ALS Pathogenesis

The molecular properties that allow UBQLN2 to act as a negative regulator of SG formation, equally promote the clearance of aggregates in C9ORF72 and FUS models of ALS [20,24,32,78]. However, ALS-causing mutations in the UBQLN2 gene, primarily at the protein’s low-complexity PXX domain [30,32], partially interfere with its ability to suppress SG formation [78]. A direct link ties UBQLN2 and HSP70, a chaperone that is central to protein disaggregation. The formation of a UBQLN2-HSP70 complex promotes aggregated protein degradation via the proteasome, including of C9orf72 dipeptides [24,25]. In addition, molecular co-chaperones of HSP70, such as BAG and DnaJ family proteins, are also involved in disaggregation of misfolded proteins in both SGs and ALS models [24,62,67]. Together, a UBQLN2-HSP70 axis serves a key disaggregase mechanism that is impaired in ALS and impact aberrant SGs or some related condensate entities that seeds the formation of ALS-associated inclusions in motor neurons. Interestingly, the proteins which are identified in ALS inclusions are more supersaturated in motor neurons than in other human cell types, pointing to a specific susceptibility of motor neurons to protein aggregation [3,4,5]. Conceivably, impaired neuronal proteostasis leads to aggregation of supersaturated proteins, however whether such supersaturated proteins accumulate into SGs remains to be determined. 

## 5. Summary

**Figure 2:**
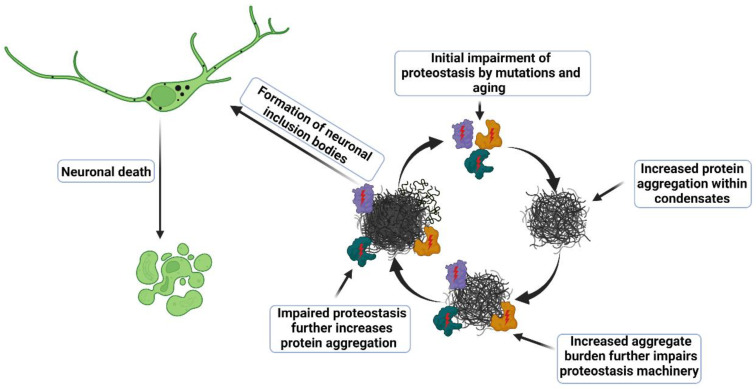
Model for dysregulated proteostasis in ALS pathogenesis. Impaired proteostasis by ALS-causing mutations, is aggravated by a decline in proteostasis capacity with aging [2], reducing neuronal capacity for handling misfolded proteins. Mutations in genes involved in protein degradation (e.g., VCP, UBQLN2, p62) may impair UPS function directly. C9ORF72-associated dipeptides inhibit the proteasome [92,93]. In addition, excess aggregation of specific mutated proteins (e.g., TDP43, FUS, SOD1, C9ORF72) exhaust and derail UPS function [94,95]. Supersaturated proteins aggregate and further contribute to proteostasis impairment. As a result of this vicious cycle, coaggregation of the neuronal proteome occurs in inclusion bodies.

The formation of protein aggregates is a pathological feature present among many neurodegenerative diseases, including ALS. Inclusions typical of ALS neuropathology and SGs share several molecular features. Decompensated proteostasis mechanisms or excess protein aggregation results in accumulation of ubiquitinated proteins into SGs, causing them to transform into an aberrant state characterized by reduced disassembly kinetics and persistence in the cytoplasm (Figure 2). Similar proteostasis abnormalities are deleterious to cells and drive nerve cell death in ALS. Future studies will likely focus on delineating the specific proteostasis factors, which either promote or inhibit protein aggregation within SGs and other condensates.

## Data Availability

All data for this review is based on publicly available literature in pubmed.
